# Low vitamin D status in nursing Pakistani mothers in an environment of ample sunshine: a cross-sectional study

**DOI:** 10.1186/s12884-018-2062-0

**Published:** 2018-10-29

**Authors:** Ghulam Mustafa, Muhammad Aslam Asadi, Imran Iqbal, Nadir Bashir

**Affiliations:** 1 Nishtar Medical university, 12-A, Gilani Colony, court road, Multan, Pakistan; 2 Department of Statistics, Bahauddin Zakariya University, Multan, Pakistan; 3 Institute of child health & children hospital, Multan, Pakistan; 4 Department of pediatrics, Nishtar Hospital, Multan, Pakistan

**Keywords:** Vitamin D deficiency, Pakistan, Vitamin D, Nursing mothers, South Punjab

## Abstract

**Background:**

The maternal 25-hydroxy vitamin D (25OHD) insufficiency is related to adverse maternal and neonatal outcome. The 25OHD content of breast milk is dependent on 25OHD status of the mothers. We undertook this study to ascertain the 25OHD status and its determinants in the nursing mothers of the south Punjab, Pakistan.

**Methods:**

We recruited 67 mothers for this cross-sectional study by convenience sampling from August 2010 to June 2011 to ascertain their serum 25OHD level & its determinants. We used SPSS 23.0 for analyses.

**Results:**

The mean age of the mothers was 25.75 ± 4.4 years. The median age (and mode) was 25 years (range 18-37 years). The majority of mothers were less than 25 years of age (62.7%), uneducated (68.7%), from rural area (70.1%), lived in open houses with ample sun exposure (85.1%) and belonged to low socioeconomic strata (71.6%).

Serum 25OHD ranged from 7.2 to 43.8 nmol/L with a mean of 20.87 ± 7.69 nmol/L. The median and mode were 21.8 nmol/L & 24.0 nmol/L, respectively. The proportion of mothers with 25OHD < 20 nmol/L (severe deficiency) was 44.8%, < 30 nmol/L (deficiency) 49.3% and < 50 nmol/L (insufficiency) 5.9%. All had 25OHD below 50 nmol/L. The oral supplementation with vitamin D (vD) was the only significant determinant of vitamin D sufficiency.

**Conclusions:**

The majority of Pakistani mothers in south Punjab are vD deficient & universal vD supplementation is the need of the hour to improve health outcomes in mothers & infants.

## Background

The maternal 25-hydroxy vitamin D (25OHD) insufficiency is related to adverse maternal and neonatal outcomes [1]. This includes gestational diabetes, preeclampsia, eclampsia, postpartum depression, low birth weight infants, type 1 diabetes, small for gestational age babies and stunted children [2, 3]. This is also associated with short and long-term consequences regarding bone health, infections, inflammatory diseases or neoplastic disorders [4]. The infants are at increased risk of 25OHD insufficiency if they are breastfeeding and not taking vitamin D (vD) supplements [5, 6]. The 25OHD content of breast milk is dependent on 25OHD status of the mother [7]. Many studies have shown that majority of the pregnant and lactating mothers have less than recommended 25OHD levels [8, 9]. The vD deficiency is rampant in womenfolk of south Asia(75-99%) where it is not expected due to ample sun exposure and lower altitude that stimulate cutaneous 25OHD synthesis [9, 10]. The mothers need much higher levels vD during lactation so that breastfeeding infants can receive appropriate amounts of 25OHD in the breast milk [6, 7, 11]. Only 55% of the infants less than 6-month age were able to achieve optimal 25OHD level with oral supplementation of the recommended 400 IU/day [12]. Therefore lactating mothers and infants need to be supplemented with vD to achieve optimal level of 25OHD in the infants [6].

In Pakistan the 25OHD levels have been found to be very low in various women populations e.g. 98.8% in female medical students of public sector hospital [13], 98.9% in premenopausal volunteers [14] and over 97% in pregnant women [15, 16]. The nursing mothers have been out of focus as a specific study group and there are no reports of 25OHD status in women of southern Punjab. This study has tried to fill this gap by reporting the results of 25OHD levels in lactating mothers and their determinants.

## Patients & methods

### Aim, design and settings

This cross-sectional study recruited mothers by convenience sampling, from August 2010 to June 2011 to ascertain the vD status of the nursing mothers and its determinants in south Punjab, Pakistan. The Multan is a major district of the south Punjab, located at almost the exact center (from North to South) of Pakistan. It lies at the height of 122 m from the sea level and is located at 30.2 N & 71.4 E. It is the 3^rd^ most populous city of the country with average income & maternal education like that of the rest of the Pakistan. The Multan features an arid climate with very hot summers (upto 54 °C) and mild winters. So, there is abundant sunshine throughout the year. The average rainfall is 127 mm. The Nishtar medical university hospital Multan is the biggest public-sector hospital (1800 bedded) catering the whole of the Southern Punjab.

### Participants

The mothers were enrolled when they came for the routine immunization of their infants (< 6 months of age) to the immunization center of the Pediatric department of the Nishtar medical university hospital, Multan. Almost equal number of nursing mothers were registered from the pediatric inpatient ward where their infants were admitted for acute respiratory problems (1-5 days of admission). The sample size 60 was calculated with 95% confidence level, 0.95 expected proportion of deficiency, 0.11 confidence interval and 1.96 standard normal deviate for alpha [17].

### Collection of data

We nominated a doctor from the department and briefed him about the objectives and methodology of taking consent, administering interview from the mothers, calculating the dietary vD intake, taking blood sample, storage, transportation, collection of results and entering the data. The doctor obtained written consent from the mothers before enrolling them in the study.

The questionnaire was prepared that included the questions seeking the demographic data about the family, mother, parity, delivery, diet, education, season of collection of blood, smoking, daily sun exposure; the characteristics of mother & household that are potentially associated with the 25OHD status. We classified socioeconomic status of the participants on the basis of monthly income as defined by Household Integrated Economic Survey of Pakistan (Low = < 6000 rupees, Middle = 6000-12,000 rupees, Upper= > 12,000 rupees) [18]. The potential sources or supplements of calcium/vD in the diet of the mother were assessed based on the reported frequency of the food items consumed over the previous week prior to the enrollment. We calculated the amount of calcium in diet by modifying the Diet History Questionnaire of the Food Frequency Questionnaire [19].

A venous blood sample of 3 ml was drawn by the standard methods [20]. It was stored in refrigerator at − 2 to − 8 degrees centigrade before transportation to the laboratory. We estimated 25OHD with (FDA approved) Abbott Laboratories’ fully automated 1-step delayed chemiluminescent microparticle immunoassay (CMIA) for 25OHD on the ARCHITECT platform (Abbott Park, IL). The serum calcium, phosphate, alkaline phosphate, parathormone and albumin were also determined.

### Outcome

The primary outcome of the study was the classification of the mothers on the basis of the mean 25OHD status. Based on current recommendations [21], the cut-off points for the vD deficiency & vD insufficiency were taken as < 30 nmol/L & < 50 nmol/L of 25OHD, respectively. The optimal level of vD was taken as > 50 nmol/L of 25OHD. Keeping in view the observed data, the mothers having 25OHD < 20 nmol/L were further classified as having severe vD deficiency. As a secondary exploratory analysis, the observations relating to the factors that potentially influenced 25OHD level were drawn for statistical evaluation as given below.

### Statistical analysis

The distribution of 25OHD among mothers (*n* = 67) is described by its mean, standard deviation (SD) and 95% confidence interval (CI) of the participants with 25OHD less than each of the cut-off values. Other than age and 25OHD, percentages of the other characteristics are provided. Two-sample independent T-test is used to compare the various determinants of the vitamin D level and their *p* values. Moreover, odds ratios (ORs) were computed to assess different potential factors for 25OHD status. All the analyses were carried out using the statistical package, SPSS 23.0. By convention, the p values of less than 0.05 were considered significant.

## Results

### Characteristics of study participants

Serum 25OHD was measured in the 67 mothers during the study. The mean age of the mothers was 25.75 ± 4.4 years. The median age (and mode) was 25 years (range 18-37 years). The majorities of mothers were less than 25 years of age (62.7%), uneducated (68.7%), came from rural area (70.1%), lived in open houses with ample sun exposure (85.1%) and belonged to low socioeconomic strata (71.6%). Only 5 women (7.5%) had completed 12 years of education (Table 1). The women were exposed (only hands and face i.e. 3-5% of the body area) to sun for 3 h daily on average for the routine household chores, usually in the morning and evening when the cutaneous synthesis of 25OHD is not very effective. They consumed around 800 mg calcium on average daily.

**Table 1:** Characteristics of the sample of nursing mothers, Pakistan

Characteristics	Mean ± SD or no.(%)
Total mothers	67
Age	
Age (years)	25.75 ± 4.4
Women age less than 20 years	12/67(17.9%)
Women age 21-25 years	30/67(44.8%)
Women age 26-30 years	16/67(23.9%)
Women age 31-40 years	9/67 (13.4%)
Housing	
Rural	47(70.1%)
Urban	20(29.9%)
Housing with Sun exposure	
Closed (without sun exposure)	10/67 (14.9%)
Open (with ample sun exposure)	57/67 (85.1%)
Socioeconomic Status	
Income less than 6000 rupees/month	48/67(71.6%)
Income between 6 and 12 thousand rupees/month	16/67(23.9%)
Income more than 12 thousand rupees/month	3/67(4.5%)
Education	
Uneducated	46/67(68.7%)
Primary (5 years of education)	9/67(13.4%)
Middle (8 years of education)	3/67 (4.5%)
Matric (10 years of education)	4/67 (6%)
Higher secondary (12 years of education)	3/67 (4.5%)
Graduate (14 years of education)	1/67 (1.5%)
Masters (16 years of education)	1/67 (1.5%)
Average daily Sun Exposure	
Daily Sun Exposure in minutes	168.81 ± 89.8
Average daily Calcium Intake	
Daily Calcium intake (mg/day)	790 ± 297.64

### vD status of nursing mothers

Serum 25OHD ranged from 7.2 to 43.8 nmol/L (n = 67) with a mean of 20.87 ± 7.69 nmol/L (95% CI: 18.9-22.7 nmol/L), the median was 21.8 nmol/L and the mode was 24 nmol/L.

**Fig. 1:**
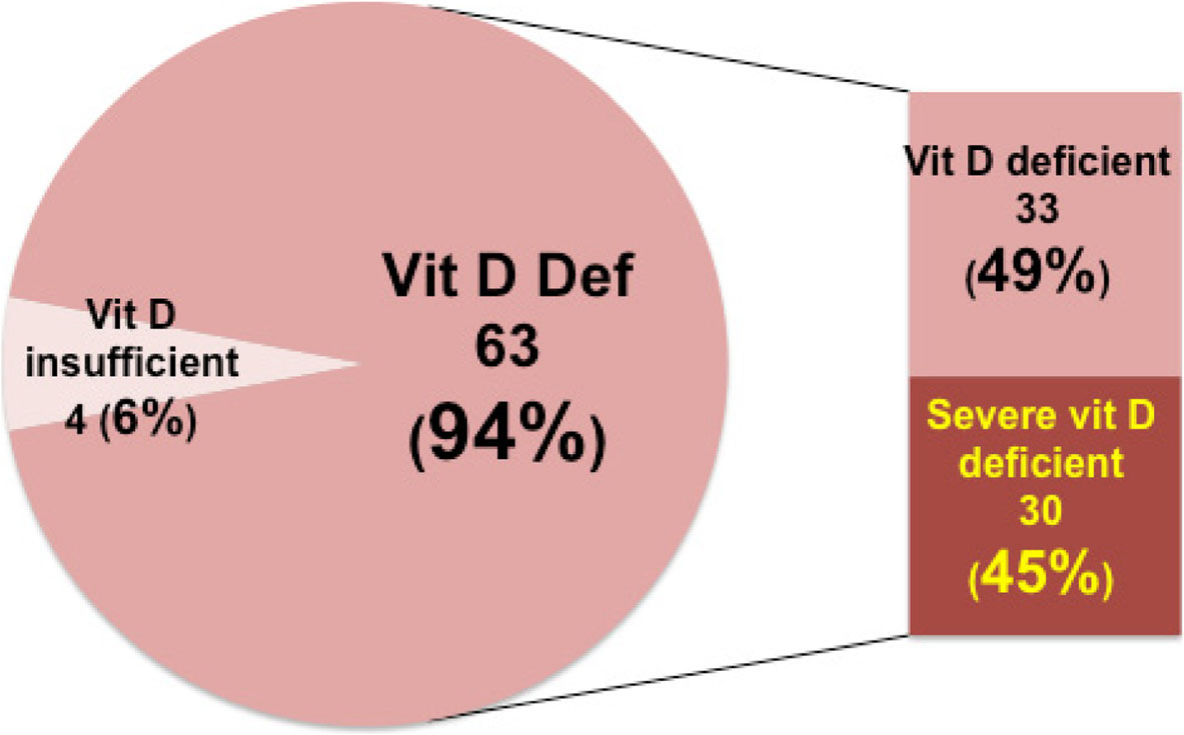
Serum vD status of the nursing mothers (percentages rounded off to nearest whole digit)

The percentage of mothers with 25OHD level < 20 nmol/L (severe deficiency) was 44.8% (95% CI: 32.6-57.4%), < 30 nmol/L (deficiency) 49.3% (95% CI: 36.8-61.8%), and < 50 nmol/L (insufficiency) 5.9% (95% CI: 1.6-14.6%) Fig. 1. All had 25OHD below 50 nmol/L.

**Fig. 2:**
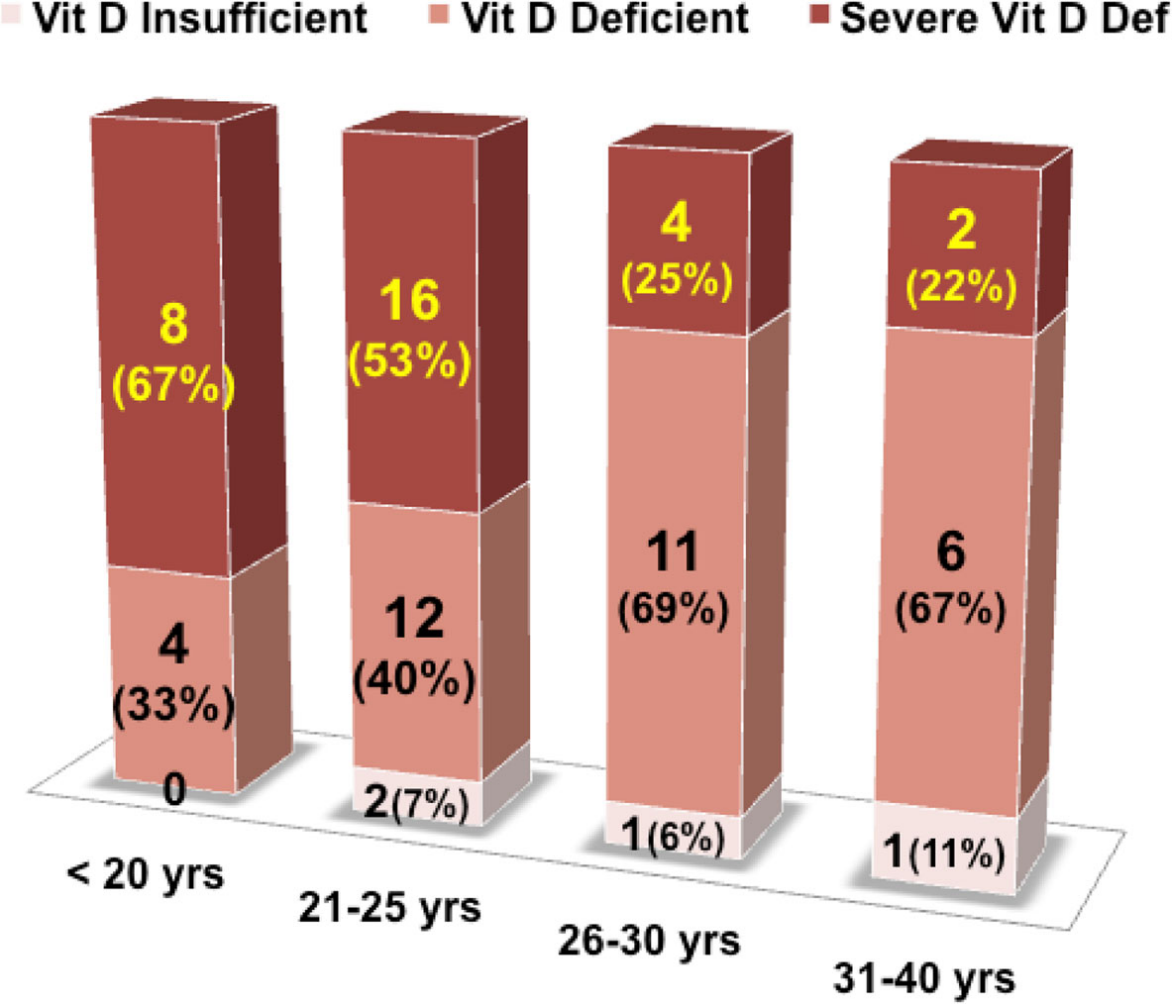
Age wise serum vD status of the nursing mothers (percentages rounded off to nearest whole digit)

The younger mothers, less than 25 years, suffered severe 25OHD deficiency more than the older age group mothers (Fig. 2). The ratio of 25OHD insufficient mothers increased with increasing age. The more mothers under 20 years are severely 25OHD deficient (66.6%). The mothers over 30 years show better 25OHD levels and less of them have severe 25OHD deficiency (22%). More mothers in this group show 25OHD levels around optimal levels (11.1%) than younger mothers.

### Characteristics associated with low vD level

We evaluated age, family income, residential area(rural/urban), education, sun exposure or vD supplementation as determinants for low 25OHD levels. We used two-sample t-test to compare the means of various determinants of low 25OHD (Table 2). The results do not show any significant difference for any factor assessed except for vD supplementation. The vD supplementation is directly proportional to the 25OHD levels.

**Table 2:** Vitamin D level with regards to various determinants (*n* = 67)

Parameters		Serum Vitamin D level (nmol/L)		*P* – value
		Mean	SD	
Age	Up to 25 years (*n* = 42)	19.70	7.97	0.108
	26 – 35 Years (*n* = 25)	22.83	6.89	
Residential status	Rural (*n* = 47)	20.49	7.86	0.538
	Urban (*n* = 20)	21.77	7.37	
Family Income	Up to Rs. 6000 (*n* = 48)	20.89	7.65	0.96
	More than 6000 rupees (*n* = 19)	20.81	7.98	
Education	Educated (*n* = 21)	23.41	6.79	0.67
	Un-educated (*n* = 46)	19.71	7.86	
Supplementation	Yes (*n* = 33)	23.35	8.20	*0.008*
	No (*n* = 34)	18.46	6.38	
Smoking environment	Yes (*n* = 35)	22.23	8.69	0.130
	No (*n* = 32)	19.38	6.21	
Vitamin D categories	Severe Vitamin D deficiency (*n* = 30)	14.26	3.69	0.000
	Vitamin D Deficiency (*n* = 37)	26.23	5.61	

## Discussion

Nowadays the vD levels are being studied all over the world and same is true for Pakistan where many reports/studies have elucidated the various aspects of its epidemiology but the vD levels in nursing mothers have been out of focus. Also, there are no studies from the south Punjab, an area where the sunlight is not only ample, but the area does usually record the highest annual temperature in the country. Despite the abundant sunlight, the findings of such a widespread vD deficiency in the nursing women is a matter of concern for all health care providers and policy makers.

Considering the deficiency & insufficiency levels of serum 25OHD 30 nmol/L & 50 nmol/L, we found that 94.1% & 5.9% of the nursing mothers were deficient & insufficient for vD, respectively. The optimal level of serum 25OHD of 50 nmol/L was not found in any mother.

Being at or near the equator it is expected that the vD level in the women residing here will be optimal without supplementation/ fortification in diet. But the evidence does not support it. Many studies [22] in the women inhabiting at or near the equator, show a high vD deficiency or insufficiency levels, quite in line with our findings.

Our data from South Punjab, Pakistan is in concordance with the findings reported from various areas of Pakistan and the region (Table 3). All studies from one end of the Pakistan to other have recorded the vD deficiency/insufficiency in over 90 to 98% of the study populations. The possible causes and determinants are unclear but might be related to the dietary patterns, study design and the geographical location. This implies that a tropical climate in itself is not necessarily protective against low vD in lactating mothers. The plausible explanations of this apparent ‘vD paradox’ in South Asia are as yet only hypothetical. The probable reasons are lack of appropriate skin exposure to sunshine during the time of ultra-violet B (UVB) radiations (11 a.m. to 3 p.m.) that activates cutaneous synthesis of precursors of 25OHD, diet that customarily does not contain daily recommended allowance and environmental pollution.

**Table 3:** Overview of studies in Pakistan & Neighbor countries for vitamin D status in women

Study	Site	Population	No.	Mean25OHD (nmol/L)^a^	Reference values of vitamin D status
Kanani et al. 2013 [13]	Karachi, Pakistan	Female medical students of Public hospital.	84	15 ± 10.71	25OHD < 25 nmol/L in 96.4% < 75 nmol/L in 98.8%
Dar et al. 2012 [14]	Karachi, Pakistan	Pre-menopausal volunteers	174	38.3 ± 15.23	25OHD < 75 nmol/L in 98.9%
Sharif et al. 2013 [15]	Lahore, Pakistan	Pregnant Lactating women	40 40	26.5 ± 17.1 21.4 ± 16.3	25OHD < 50 nmol/L in 95% 25OHD < 50 nmol/L in 97.5%
Aslam et al. 2012 [16]	Faisalabad, Pakistan	Pregnant women	61	NA	25OHD < 75 nmol/L in 97%
Junaid et al. 2015 [24]	Lahore, Pakistan	Child bearing age working women	215	40.4 ± 34.45	25OHD < 50 nmol/L in 73% 25OHD < 75 nmol/L in 90%
Kaykhaei et al. 2011 [25]	Zahedan, Iran	Adult males 431 Adult females 562	993	34.3 ± 29.43	25OHD < 75 nmol/L in 94.7%
Sharma et al. 2016 [26]	Delhi, India	Pregnant women	418	38.5 ± 22.39	25OHD < 80 nmol/L in 93.5%
Salameh et al. 2016 [27]	Doha, Qatar	Exclusively breast-feeding mothers	60	NA	25OHD < 50 nmol/L in 78%
Ullah et al. 2013 [28]	Dhaka, Bangladesh	Pregnant women	188	61.2 ± 1.78	25OHD < 75 nmol/L in 78%
Haugen et al. 2016 [29]	Kathmando, Nepal	Nursing mothers	500	47.4 ± 16.4	25OHD < 50 nmol/L in 73.8% 25OHD < 75 nmol/L in 95.2%
Fouda et al., 2017 [30]	Riyadh, Saudi Arab	Pregnant women	1097	31.2 ± 20.90	25OHD < 50 nmol/L in 84.3% 25OHD < 75 nmol/L in 94.2%
Zhau et al., 2017 [9]	Beijing, China	Lactating women	2004	NA	25OHD < 50 nmol/L in 97.9% 25OHD < 75 nmol/L in 99.7%

^a^*25OHD* 25 hydroxy vitamin D, @ = Not available

It is realistic to believe, keeping the above factors of vD deficiency correlates in mind, that the vD deficiency can be speculated in our community if they are not taking supplements. The trends in lifestyle, the clothing, season, outdoor activities, patterns of rearing & dietary practices of the rest of the community women are almost similar to the one studied. So, it can be deduced that vD deficiency is widespread in our communities. These realities are more relevant for the mothers who have to breastfeed their children. It has been shown that the diet of low-income group mothers is low in calcium and high in phytates that leads to impaired absorption of calcium and generates increased demands of 25OHD [23]. As the maternal vD status determines the concentration of vD metabolites in breast milk so ongoing deficits in postnatal infants’ vD intake would be influenced by maternal vD levels [5–7]. Since the diet of the mothers is very poor source of vD, therefore interventions must be planned to supplement their diet to improve the situation [5, 11]. Further studies may be planned to find out the best suitable interventions & plan of fortification. However, rigorous studies of the broad health benefits of interventions to improve the antenatal or postnatal vD status in South Asian mothers and infants have yet to be testified.

We have not been able to find out the exact determinants of vD deficiency as is evident from the Tables 2, 4. The age, residential status in the rural/urban area, family income, maternal education and smoking environment do not show any significant effect on the 25OHD levels of the mothers. The women in the study attired the similar way so we did not study it as a variable. There was no difference in the exposed area of the body in the females. Barely 3-5% body area was exposed (face & hands) to the sun in the open houses. The odds ratio analysis of the vD deficiency determinants show that only vD supplementation is the significant factor. The mothers taking vD supplements show better vD status than those who are not taking vD supplements. Given the dynamics of the vD synthesis and deficiency determinants it seems a reasonable inference. This clearly underscores the need for the vD supplementation in our populations.

**Table 4:** Odds ratio for various factors of vD status

		Vit D Status			
Categories	Classification	Severe Vitamin D Deficiency	Vitamin D deficiency	OR	*p*-Value
Age group	Up to 25 yrs	24	18	4.22	0.0105
	> than 25 yrs	6	19		
Residential Status	Rural	23	24	1.78	0.2964
	Urban	7	13		
Level of education	Educated	5	16	0.26	0.0228
	Uneducated	25	21		
Supplementation	Yes	13	20	0.65	0.3837
	No	17	17		
Smoking environment	Yes	15	20	0.85	0.7412
	No	15	17		

### Limitations

Our study is limited by its restricted geographic scope, sample size and cross-sectional design. Otherwise, the community based random sampling is fairly depictive of the trends in the general population regarding vD status and its determinants. The health status of the mothers may impact vD status, but we did not have access to this information.

## Conclusions

Our study provides initial observations on the 25OHD status of nursing mothers in south Punjab, Pakistan. The situation is very alarming as nearly, all the mothers were either deficient or insufficient for 25OHD. The relative, left-shifted distribution of vD in this study sample is likely representative of the broader population. This may also be associated with an excess burden of rickets, symptomatic hypocalcemia, growth faltering, or extra-skeletal health outcomes [1, 3]. Therefore, recommendations for universal vD supplementation in Pakistan is not untimely. The nursing mothers need to be supplemented with vD to provide more 25OHD in breast milk. This may help the infants to achieve optimal 25 OHD level with oral supplementation [6]. Needless to say, that the causes and consequences of low vD in nursing mothers in South Asia need to be investigated further.

## Abbreviations

25OHD: 25-hydroxy vitamin D
CI: Confidence interval
CMIA: Chemiluminescent Microparticle Immuno-Assay
IU: International Unit
nmol/L: nano-mole/Liter
ORs: Odds ratio
SPSS: Statistical Package for Social Sciences
UVB: Ultra-violet B
vD: vitamin D
